# Eye movement instructions modulate motion illusion and body sway with Op Art

**DOI:** 10.3389/fnhum.2015.00121

**Published:** 2015-03-26

**Authors:** Zoï Kapoula, Alexandre Lang, Marine Vernet, Paul Locher

**Affiliations:** ^1^IRIS Team, Physiopathology of Binocular Motor Control and Vision, Neurosciences, UFR Biomédicale, CNRS, University Paris VParis, France; ^2^Montclair State UniversityUpper Montclair, NJ, USA

**Keywords:** posture, posturography, macro and micro eye movements, saccade, vergence, motion sensation, movement sensation

## Abstract

Op Art generates illusory visual motion. It has been proposed that eye movements participate in such illusion. This study examined the effect of eye movement instructions (fixation vs. free exploration) on the sensation of motion as well as the body sway of subjects viewing Op Art paintings. Twenty-eight healthy adults in orthostatic stance were successively exposed to three visual stimuli consisting of one figure representing a cross (baseline condition) and two Op Art paintings providing sense of motion in depth—Bridget Riley’s *Movements in Squares* and Akiyoshi Kitaoka’s *Rollers*. Before their exposure to the Op Art images, participants were instructed either to fixate at the center of the image (fixation condition) or to explore the artwork (free viewing condition). Posture was measured for 30 s per condition using a body fixed sensor (accelerometer). The major finding of this study is that the two Op Art paintings induced a larger antero-posterior body sway both in terms of speed and displacement and an increased motion illusion in the free viewing condition as compared to the fixation condition. For body sway, this effect was significant for the Riley painting, while for motion illusion this effect was significant for Kitaoka’s image. These results are attributed to macro-saccades presumably occurring under free viewing instructions, and most likely to the small vergence drifts during fixations following the saccades; such movements in interaction with visual properties of each image would increase either the illusory motion sensation or the antero-posterior body sway.

## Introduction

It has been shown that the study of postural adjustments of the body (posturography) to pictorial elements and the overall compositional organization of artworks can provide a physiological measure of a viewer’s reaction to these stimulus properties. For example, Kapoula et al. (2011) investigated the effects of pictorial depth on body sway. They found a significantly greater increase in sway when observers untrained in the visual arts fixated the principal background area vs. the foreground of the Renaissance painting *The Annunciation* by Piero della Francesca. Additionally, the researchers observed significant differences in postural sway when viewers examined two abstract paintings by the artist Maria Helena Vieira Da Silva; greater sway was recorded for the painting *O Quatro Cinzento* which contains a greater intensity of visual depth cues than the second work viewed entitled *Egypt*. The effect was lessened when participants viewed experimentally generated cubist transformations of the same paintings in which pictorial depth cues were neutralized. Various potential mechanisms on the effect of body sway have been discussed: explicit motor correlate of the perception in depth, or mental simulation of maneuvering the body in the virtual pictorial space. Another, simpler mechanism, privileged by the authors, was the direct influence of pre-attentive visual analysis of depth cues on body sway rather than the conscious perception processes mentioned above.

In another posturography study, Nather et al. (2010) investigated the ability of the depiction of body movement in static images to generate body sway in observers. University students untrained in the visual arts or classical dance viewed photographic images of two sculptures of ballerinas created by Degas. One ballerina was in a static “standing” position and the other was depicted taking a large step that appeared to place the dancer in a state of potential imbalance. Participants, who stood on a force plate to measure body sway, observed each image as they estimated its presentation time duration. Nather et al. found that participants exhibited significantly greater body sway when they observed the dancing ballerina than the static one demonstrating that images of body movement internally generate unconscious body oscillations.

Kapoula and Gaertner (2015; see also Locher and Kapoula, 2012) reported that depicted motion cues and the structural organization in paintings have the ability to physically move viewers during an aesthetic experience with art and that the neural mechanisms responsible for body sway are differentially sensitive to the strength and lateral organization of motion cues in paintings. Their art stimuli consisted of two “matched” compositions by Monet that contained movement cues produced by the wind and the mirror-image or reversed-view versions of the originals. The paintings, entitled in English *Study of a Figure Outdoors—Facing Right* and *Study of a Figure Outdoors—Facing Left* (the original titles are *Essai de Figure en plein air—Femme à l’ombrelle tournée vers la droite* and *Essai de Figure en plein air—Femme à l’ombrelle tournée vers la gauche*, respectively), are very similar in overall structural content and organization, color, and size. In both works the wind is depicted as blowing from right to left, as indicated by movement cues in the clouds, the woman’s dress and scarf, vegetation, etc. However, in one version the woman is facing into the wind and in the other version she has her back to the wind. Image cues suggest a stronger perceived effect of the wind in the painting in which the woman is facing into the wind. University students with no training in the visual arts stood on a force pad that provided measures of postural body sway as they viewed actual-size images of the paintings.

Analyses revealed differential effects for body sway measures between the two paintings as a function of their depicted depth cues, overall structural arrangement, and lateral organization. Specifically, the surface area of body sway (surface of the excursions of center of body pressure measured with the confidence ellipse including 90% of all sampled positions) and the variance of the speed of body sway were each found to be greater, on average, when the painting *Study of a Figure Outdoors: Facing Left* was viewed in mirror orientation than when it was projected normally. This indicates that the mirror image of this picture was more destabilizing than when it was seen in the normal orientation. No such effects were observed for participants who viewed *Study of a Figure Outdoors: Facing Right* in both normal and mirror orientations. These results provide evidence that people can be physically moved when viewing representational art and that the mechanisms responsible for postural stability are differentially sensitive to depicted motion cues and their lateral organization in paintings.

The graphic and compositional devices used by artists to depict motion in representational and some types of abstract art are rapidly captured by the observer (see Cutting, 2002, for a description of these techniques). The evocativeness of these motion cues is based upon one’s knowledge of the visual world. The present study concerns Op Art, a style of abstract art in which lines, forms and space are organized in such a way as to provide different types of optical illusions, e.g., movement, hidden images, patterns, vibration, etc. Here we are interested on movement illusion and particularly movement in depth. Motion sensed in Op Art may be considered an autonomous visual attribute (Zeki, 1999) that is the product of a “responsive eye” rather than requiring information processing. Many possible causes of this motion illusion in Op Art have been proposed and investigated, including the involvement of microsaccades (Zanker and Walker, 2004; Hermens and Zanker, 2012). Additionally, Zanker and Walker note that image instability due to large “conventional” saccadic eye movements may also contribute to this motion illusion.

The goal of the present research was to determine whether instructions on eye movements (fixating vs. free viewing) would influence the sensed motion in Op Art and would have an impact on a viewer’s body sway. The question is part of the new research field we call neurophysiology of aesthetics focused on sensorimotor mechanisms of interaction between the observer and the artwork, in line with theories of embodied cognition (see Wilson, 2002).

To study this question, we used as stimuli two Op Art paintings that provide the 3D appearance of movement in depth, namely *Movement in* *Squares* by Bridget Riley and *Rollers* by Akiyoshi Kitaoka. Research has shown postural adjustments with body sway in the anterior-posterior axis when individuals view moving patterns in the depth axis (e.g., Kuno et al., 1999; Guerraz et al., 2001). Additionally, Kapoula et al. (2011) have shown body sway in the anterior-posterior axis while participants viewed paintings with a strong perspectivist construction giving rise to vivid depth perception. Thus, it is reasonable to expect similar effects with illusory motion in depth. To examine the impact of eye movements on viewers’ body sway, participants in the present study examined each art work under two conditions; they either were instructed to fixate it without moving their eyes or they were free to visually explore the painting. If saccadic eye movements contribute to the strength of the motion illusion in Op Art, motion illusion and perhaps body sway measures should be stronger for the free viewing vs. the static, fixation condition.

## Method

### Participants

Participants consisted of 28 volunteer adults (*M* = 33.7, *SD* = 13.9 years of age) who had no training in the visual arts. They all demonstrated high visual acuity (20/25 or better, Snellen notation) and none reported suffering from neurological disease, or muscular, vestibular, visual or any other known abnormalities.

### Stimuli and Apparatus

**Figure 1:**
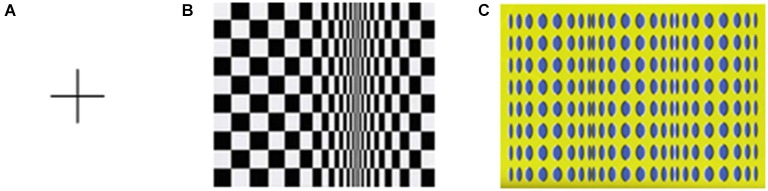
**Cross figure (A) for baseline condition and Riley’s *Movement in Squares* (B) and Kitaoka’s *Rollers* (C) paintings**.

Stimuli consisted of one figure composed of a cross, i.e., two perpendicular lines measuring 40 cm each, i.e., ~11.4° of visual angle (baseline condition) and two Op Art paintings–Bridget Riley’s (1961) *Movement in Squares* and Akiyoshi Kitaoka’s (2004) *Rollers*. Figure 1 contains an image of each stimulus. The original Op Art paintings measure 122 × 122 cm and 131 × 92 cm, respectively. These two paintings were chosen because both images provide the 3D appearance of movement in depth as indicated by a preliminary study, in which observers indicated a strong sensation of movement in both artworks. In addition, in the present study the participants indicate comparable illusory motions for these two artworks (see below). Furthermore, Kitakoa’s painting was used because there is scientific work on the psychophysical properties of this stimulus and hypotheses on the mechanisms explaining motion sensation including the role of eye movements (see Fermüller et al., 2010). Additionally, many of Bridget Riley’s Op Art paintings have been used as stimuli in a number of investigations of movement perception and optical illusions (see, e.g., Nather et al., 2013).

Posture was measured for 30 s per condition using a body fixed sensor (accelerometer). The accelerometer (Dynaport, MiniMod, McRoberts B. V. The Hague, The Netherlands) was placed at a participant’s lower back (L5). The MiniMod makes use of a triaxial scismic acceleration sensor (AXXL202, Analogue Devices, Norwood, MA, USA). The sensor’s full scale range is ± 2 degrees. The frequency response of the sensor is 0–6 Hz. (3 dB). The sampling frequency is set to 100 Hz.

The system generates the following parameters: (1) the normalized area, i.e., the body sway area divided by the duration of the recording (in mm^2^/s) obtained after integration of acceleration signal, for more details see Lindemann et al., 2012; (2) the Root-Mean-Square of Medio-Lateral body sway (RMS of M/L in mm); (3) the Root-Mean-Square of Antero-Posterior body sway (RMS of A/P in mm); (4) the RMS of M/L velocity (in mm/s); (5) the RMS of A/P velocity as well as (6) the mean power frequency (Hz). The first three measures describe the viewer’s stability while the last three concern mostly the energy used to stabilize the body.

A pilot new study provided eye movements recordings for a single-subject. Eye movements were recorded with the eyeseecam device at 220 Hz.^1^

### Procedure

The investigation adhered to the tenets of the Declaration of Helsinki. Informed consent was obtained from each participant after the nature of the experiment and apparatus was explained to her or him. Participants were then randomly divided into two groups. In both groups, they were successively exposed to the cross figure (baseline condition) and then to the two Op Art paintings in a random order; the viewing periods lasted 30 s each and were separated by a short break (less than a minute). In the baseline condition, subjects were asked to fixate the center of the cross; the purpose of the baseline condition was to show that there was no inherent differences in body sway between the two groups. The order of the two paintings was counterbalanced among subjects. Before each Op Art painting projection, participants were instructed either to look at the picture freely (group 1) or to stare at the center of the picture (group 2). Participants viewed the paintings projected in their actual sizes; the distance between painting and observer was 2 m. After exposure to each stimulus, participants indicated their sensation of movement illusion on a 0–10 scale–the value 0 corresponded to a sensation of the complete absence of movement while the value 10 corresponded to the maximal movement illusion. The purpose of this evaluation was to quantify subjective motion sensation and to cross it with results from posturography. Also, we used a design with two different groups of subjects in order to avoid possible effects from seeing the same painting twice.

## Results

Because of the relatively small size population, and the absence of normal distribution for some variables (e.g., normalized area, RMS of A/P body sway, RMS of A/P velicity, *p* < 0.05 with the W test of Shapiro-Wilk), non-parametric tests were used.

### Subjective Evaluation Motion Illusion

**Figure 2:**
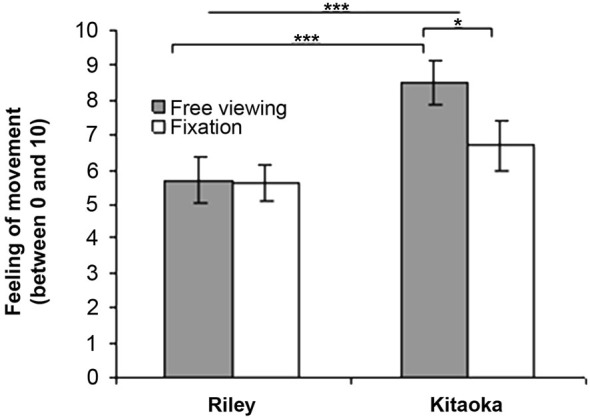
Subjective evaluation (mean and standard error) of motion illusion when looking at Riley’s and Kitaoka’s paintings in free viewing and fixation conditions (*p < 0.05; ***p < 0.001).

The results for the subjective evaluation of the sensation are presented in Figure 2 as a function of the stimulus (Riley’s and Kitaoka’s paintings) and viewing instructions (free viewing vs. fixation). For the comparison in terms of the viewing conditions, the data were analyzed using a Mann-Whitney *U*-test. As illustrated in Figure 2, the sensation of movement was significantly higher in the free viewing condition than in the fixation condition for Kitaoka’s painting (*U* = 50, *p* = 0.027). This difference was however not significant for Riley’s painting (*U* = 94.5, *p* = 0.87). The subjective evaluation data were also analyzed in terms of stimuli using a Wilcoxon test. The sensation of movement was significantly higher during the projection of Kitaoka’s painting than Riley’s work (*Z* = 3.85, *p* = 0.0001) and this difference was found to be significant in the condition with instruction for free viewing (*Z* = 3.47, *p* = 0.0005) but not in the fixation condition (*Z* = 1.66, *p* = 0.10).

### Posture Data in the Baseline Condition

The baseline condition (the cross image) data for the two groups of subjects for each body sway variable were compared. Table 1 reports the mean values and standard deviations for both groups as well as Mann-Whitney U test result and the *p*-value for each variable. As shown in this table, no differential effect was found for any body sway variable. Therefore, the postural performances of the two groups were similar in the baseline condition. This is important as the next sections present posture data for the two groups seeing the paintings with different instructions (fixating vs. exploring). This design was used to avoid any learning effect due to multiple presentations of a given painting.

**Table 1:** **Body sway variables mean values and standard deviations for the baseline condition, and Mann-Whitney *U* and *p*-values comparing both groups of participants body sway variables**.

	Group 1	Group 2
	*Mean (SD)*	*Mean (SD)*	*U*	*p-value*
Normalized Area (mm^2^/s)	2.18 (1.16)	1.75 (0.89)	68.5	0.275
RMS A/P Body Sway (mm)	4.67 (1.74)	4.41 (1.68)	82.0	0.462
RMS M/L Body Sway (mm)	1.47 (0.59)	1.26 (0.43)	69.5	0.442
RMS A/P Velocity (mm/s)	19.36 (9.03)	17.57 (5.75)	83.0	0.959
RMS M/L Velocity (mm/s)	6.36 (2.27)	6.42 (2.69)	70.0	0.908
Mean Power Frequency (Hz)	6.93 (1.56)	7.19 (1.00)	82.5	0.918

### Posture Data While Viewing the Op Art Paintings

**Figure 3:**
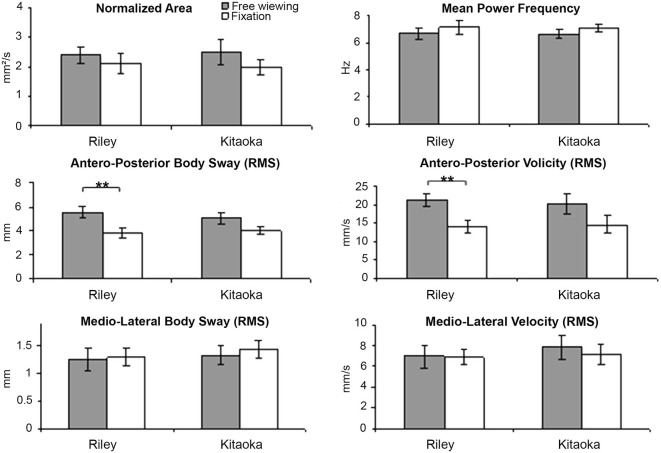
Body sway variables’ mean values and standard error for Riley’s and Kitaoka’s paintings in both viewing conditions (free viewing vs. fixation) (**p < 0.01).

Means and standard errors for the six body sway variables are shown in Figure 3 as a function of the two artworks seen in both viewing conditions. These values are also summarized in Table 2. All body sway parameters for Riley’s and Kitaoka’s paintings were compared with a Wilcoxon test that revealed no statistically significant differences (Wilcoxon test, all *z* < 0.94; *p* > 0.35); as can be seen in Figure 3, any two black bars are very similar, and any two white bars are also very similar. These data were also compared as a function of viewing conditions (free viewing vs. fixation) using a Mann-Whitney *U*-test. As illustrated in Figure 3, the antero-posterior body sway for the Riley painting was found to be significantly higher in the free viewing condition than in the fixation condition, and this was the case both in terms of amplitude and velocity (*U* = 29 and 36, respectively, *p* = 0.0035, after Bonferroni correction *p* = 0.021). Interestingly, the difference was significant for both antero-posterior variables for Riley’s painting but failed to achieve significance for Kitaoka’s painting. However, it should be noted that, as seen in Table 2, antero-posterior body movement was somewhat higher, on average, for both paintings compared to medio-lateral body sway. Antero-posterior movement is indicative of the illusion of movement in depth and many participants during debriefing agreed that the images, especially the work by Kitaoka, produced a sense of motion in depth. All other comparisons for the remaining postural parameters shown in Table 2, were not significant (*U* > 45, *p* > 0.076, after Bonferroni correction *p* > 0.45).

**Table 2:** **Body sway variables mean values and standard deviations for Riley’s and Kitaoka’s paintings in both viewing conditions (free viewing vs. fixation)**.

	Free viewing condition		Fixation condition	
Body Sway Variables	Riley	Kitaoka	Riley	Kitaoka
Normalized Area (mm^2^/s)	2.41 (1.23)	2.51 (1.28)	2.11 (1.64)	1.99 (1.37)
RMS A/P Body Sway (mm)	5.55 (1.49)	5.04 (1.37)	3.79 (1.17)	4.02 (1.72)
RMS M/L Body Sway (mm)	1.25 (0.65)	1.33 (0.67)	1.29 (0.50)	1.43 (0.57)
RMS A/P Velocity (mm/s)	21.29 (8.83)	20.26 (7.36)	13.96 (8.31)	14.56 (6.29)
RMS M/L Velocity (mm/s)	6.97 (3.90)	7.82 (3.87)	6.90 (4.39)	7.19 (4.42)
Mean Power Frequency (Hz)	6.72 (1.91)	6.67 (1.29)	7.15 (1.17)	7.11 (1.30)

Finally, comparisons of postural parameters between the fixation condition and the fixation of the cross (baseline condition) showed no significant differences for any of the parameters (Wilcoxon test, comparison data from group 2, baseline vs. Riley (fixation instruction) and baseline vs. Kitaoka (fixation instruction), all *z* < 1.85; uncorrected *p* > 0.06).

### Correlation Between Subjective Sensation of Motion and Body Sway

Correlation values between subjective sensation of motion and any of the body sway parameters (RMS A/P, RMS M/L, RMS A/P velocity, RMS M/L velocity, Mean Power Frequency) all failed statistical significance (*p* > 0.05).

## Discussion

### Eye Movement Instructions Enhance Motion Sensation for Kitaoka’s Artwork

**Figure 4:**
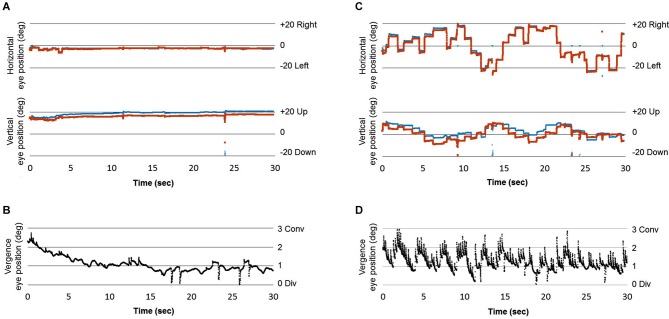
**(A)** Horizontal and vertical position of the right and left eye while fixating Kitaoka’s image, **(B)** Difference between the horizontal left and horizontal right eye position indicating the vergence change while fixating Kitaoka’s image; positive inflection indicates a small convergent drift of the eyes during the fixation period interrupted by small divergence periods, **(C)** Horizontal, and vertical position signals from left and right eyes during free viewing instruction of Kitaoka’s image; large saccades (mostly horizontal) are made, **(D)** Vergence signal during saccades and fixations between the saccades during free viewing instruction of Kitaoka’s image. During the saccades substantial divergent transients occur (downward inflexion), while during subsequent fixations convergent drifts occur that are, at least qualitatively, of higher velocity than those during fixations. Eye movements were recorded with the eyeseecam device at 220 Hz.^2^

This study examined the subjective sense of motion and body sway in relation to eye movement instructions to two Op Art stimuli previously shown to generate strong motion perception in a pilot study. Motion sensation was rated as moderate to high for the Riley and Kitaoka paintings, respectively. Furthermore, ratings for Kitaoka’s image were significantly higher in the condition with free viewing instruction, which presumably involved large saccades compared to the fixation condition for the same artwork. Eye movements were not recorded in this study; yet, instructions for free viewing presumably cause saccades to different parts of the image (a single subject eye movement recording from a pilot new study is shown in Figure 4). To our knowledge, this is the first time such an effect of eye movement instructions has been reported. Research by Zanker and Walker (2004), although highlighting the important contribution of eye movements to illusory motion in Op Art, focused on micro-movements that are known to occur during fixations. Here we show that instructions for free viewing that naturally involve macro-movements, can contribute even more to such sensation.

### Saccades Vergence and Motion Processing—A Reconciling Interpretation

One of the first interpretations proposed for the role of eye movements for movement illusion with Op Art (see Campbell and Robson, 1958) concerned the accommodation of the eyes. Accommodation of the eyes induces vergence eye movements because of the natural accommodation vergence link; reciprocally vergence eye movements can modulate slightly the accommodation of the eyes (see Leigh and Zee, 2006). Every single macrosaccade we make while exploring images (even when an image is presented at frontoparallel plane) is accompanied by a small transient vergence of the eyes (mostly divergence) which is followed by restoring vergence drift (mostly convergence) that occurs during the subsequent fixation, namely during the first 48 ms of the fixation. This pattern of intra-saccadic transient divergence followed by convergent post-saccadic fixation drift is due to the fact that one eye (most frequently the abducting eye, the one moving outwards) makes a slightly larger saccade than the other. It occurs similarly during reading and during exploration of images (see Vernet and Kapoula, 2009). We suggest that the motor mechanism contributing to movement illusion with the images we used in our study is precisely this vergence instability, particularly the drifting vergence during the early part of each fixation.

Figure 4 shows preliminary results of eye movement recording from one subject while viewing Kitaokas’ artwork under fixation **(A)** and free viewing conditions **(C)**. In the latter case large, mostly horizontal saccades are made (see **C**); the trace indicating the vergence change during the saccade and during the fixation periods between saccades shows larger vergence changes in the free viewing condition than in the fixation condition (see **D** vs. **B**). These preliminary observations support our interpretations. Further research with simultaneous eye movement and posture sway recordings combined with explicit instructions for eye movements in depth (e.g., convergence vs. divergence, see below) is needed, as well as further technical developments to correlate over time antero-posterior body sway and velocity of vergence drifts during fixations.

### Interplay Between Paintings, Eye Movements and Body Sway

The second major finding of this study is that the instruction for free visual exploration of the two Op Art paintings induced a larger antero-posterior body sway both in terms of speed and displacement in the free viewing condition as compared to the fixation condition. The difference between conditions was statistically significant for the Riley painting. The specificity of this effect (in terms of antero-posterior axis) is highly plausible and relevant, as the paintings caused motion sensed in depth. (An interesting experiment would be to investigate whether Op Art causing lateral motion sensation induces lateral body sway.) Thus, body sway seems to reflect specific aspects of visual psychophysics of the paintings giving rise to the sensation of motion in depth.

We believe that the particular visual properties of these two works induced a sense of motion and body sway in the antero-posterior axis. Eye movements, particularly the naturally occurring small vergence eye movements during saccades (both regular saccades and microsaccades) and most important vergence drifts during fixations in interplay with the visual properties of these images, could be the mechanism mediating the motion illusion. Body sway in the antero-posterior axis in turn can also induce or accentuate small vergence eye movements in depth that could further enhance movement illusion in depth. Thus reciprocal interactions could operate between body sway and eye movements in depth, both stimulated by specific visual properties of these images. Dissociating between the two components is practically impossible and this is the case for all studies on postural control; control of posture being an active mechanism involving continuous integration and interactions between visual, eye movement, proprioceptive, vestibular and somatosensory signals. As mentioned in the Introduction, laboratory studies have shown postural adjustment with body sway in the depth axis when viewing moving patterns in the depth axis (see Kuno et al., 1999; Guerraz et al., 2001).

### Stronger Motion Sensation for Kitaokas’s Artwork but Stronger Body Sway for Riley’s Artwork

As discussed above, motion illusion was higher for Kitaoka’s artwork compared to that by Riley, particularly in the condition with free viewing instruction. Higher sensation for the former could be attributed to the effect of repeating patches which have an asymmetric intensity profile creating very strong illusory effect (see Fermüller et al., 2010). These compositional techniques were specifically used in the painting *Rollers* employed in this study, inducing illusory rotation of three tubes in the antero-posterior plan. Yet, in terms of effective anterior/posterior body sway, the enhancement when free viewing was instructed compared with the fixation condition, although present, was of a lesser degree than for Riley’s artwork (see Figure 3). Thus, enhancement in sensation and enhancement in body sway due to instructions for free viewing seem to operate differentially for the two artworks. In this respect, there might be no link between subjective reports of motion illusion and effective body motion control. Weak correlations between reports of motion sensation and physiologic measures have been reported before (see Kapoula et al., 2011; Kapoula and Gaertner, 2015). A tentative explanation of the differential effects of eye movement instructions on body sway vs. motion illusion for the Riley’s vs. Kitaoka’s image follows: for the Kitaoka’s image visual features provide sense of forecoming (toward the observer) motion and would be congruent with the known convergent drift occurring just after saccades (see abduction adduction asymmetry causing the eyes to diverge during the saccade and converge during the fixation); the two together reinforce the sense of proximal motion that perhaps prevents significant increase of antero-posterior body sway. In general, sense of proximity is associated with postural stability (see Kapoula and Lê, 2006; Kapoula et al., 2011). In contrast, for Riley’s image, visual cues provide far-going distal motion illusion opposing to convergent drift of the eyes. Sensation of motion illusion is not enhanced in this case; the distal motion illusion and incongruence however could enhance body sway significantly. This hypothesis can be tested with further studies involving further instructions for different types of eye movements, including convergence and divergence. We hope that the present, preliminary study will stimulate further research.

## Conclusion

In conclusion, this empirical investigation opens the posturography field to the study of motion illusion in Op Art and raises new questions on cross interactions between eye movements and body sway control. Objective binocular eye movement recording and analysis in high temporal resolution of small vergence drifts combined with posturography can provide new light on physiological mechanisms. The results support the view that the motion sense created by Op Art is generated by eye movements, and this in the present study translates to influence on body sway itself.

## Conflict of Interest Statement

The authors declare that the research was conducted in the absence of any commercial or financial relationships that could be construed as a potential conflict of interest.

## Footnotes

^1^http://eyeseecam.com/

^2^http://eyeseecam.com/
